# Fluid–Thermal–Structure Coupled Analysis on the Tempering Characteristics of Glassware During Air Cooling

**DOI:** 10.3390/ma19132794

**Published:** 2026-07-01

**Authors:** Kang An, Hao Zheng, Chi Qin, Pengfei Zhang, Yajing Zhang, Wenbin Dong

**Affiliations:** College of Intelligent Manufacturing, Anhui Science and Technology University, Chuzhou 239000, China; ankang@ahstu.edu.cn (K.A.); qinchi@ahstu.edu.cn (C.Q.); zhangpengfei@ahstu.edu.cn (P.Z.); zhangyj@ahstu.edu.cn (Y.Z.)

**Keywords:** glassware, physical tempering, fluid–thermal–structure coupling, residual stress, heat transfer uniformity, numerical simulation

## Abstract

Physical tempering is widely used to enhance the mechanical strength and thermal stability of glassware. Traditional numerical studies commonly adopt the uniform heat transfer coefficient assumption, which significantly deviates from the actual non-uniform jet cooling conditions, especially for glassware with complex three-dimensional curved surfaces. In this work, a fluid–thermal–structure sequential coupling numerical model for low-borosilicate glassware was developed using STAR-CCM+. The Realizable k-ε turbulence model, temperature-dependent thermophysical properties of glass and air, and transient non-uniform convective heat transfer boundaries were employed. Flow characteristics, heat transfer behavior, and residual stress distribution during air cooling were systematically investigated. The simulation results were verified using a polarizing stress instrument. Results indicate that obvious flow separation and vortices occur at the curved regions, resulting in highly non-uniform heat transfer. Temperature uniformity first decreases and then rebounds, while stress uniformity finally stabilizes above 90%. The through-thickness stress exhibits a parabolic profile with surface compression and internal tension. The maximum relative error between simulation and experiment is below 6%, demonstrating the reasonable engineering accuracy of the sequential coupling framework. Ultimately, these numerical observations quantify the fluid–thermal–structural interactions and underscore the critical importance of integrating realistic non-uniform aerodynamic boundaries.

## 1. Introduction

The physical tempering process is widely applied to enhance the mechanical strength [1,2,3,4] and thermal shock resistance of glass products [5,6,7]. In aerodynamic tempering, glassware such as low borosilicate glass is heated and subsequently rapidly quenched using high-velocity air jet arrays [8]. The significant temperature gradient generated during this process induces residual compressive stresses on the glass surface and tensile stresses in the interior [9]. However, precisely controlling the convective heat transfer conditions during the cooling stage—ensuring the required temperature gradient for tempering while preventing glass fracture caused by excessive initial transient tensile stress—is the core key to improving production yield [10,11,12].

The prerequisite for precisely controlling convective heat transfer is the accurate prediction of residual stress, which relies on the precise modeling of both the material’s internal structural field (e.g., relaxation mechanisms) and the external thermal field. Regarding the structural field, since Tool [13] proposed the concept of “fictive temperature,” the TNM structural relaxation model derived by Narayanaswamy [14] et al. has achieved the quantitative description of glass volume relaxation and stress evolution, becoming the classical constitutive framework. Subsequently, scholars introduced relaxation theories into spatial numerical solutions. For instance, Daudeville [15] and Wu [16,17] et al. employed thermal–structural coupled finite element analysis to reveal the dynamic evolution mechanism of internal stresses in glass driven by transient temperature gradients.

Although the aforementioned fundamental theoretical studies have made significant contributions, they mostly adopted the idealized assumption of a “uniform convective heat transfer coefficient,” neglecting the dynamic characteristics of the local flow field. Consequently, scholars such as Iglesias [18] and Pourmoghaddam [19] introduced computational fluid dynamics [20] (CFD) to investigate the actual impact of non-uniform air cooling on the heat transfer and stress fields. However, a critical scientific gap remains: the majority of these advanced fluid–thermal–structural coupling simulations still primarily focus on flat architectural or automotive glass (including those with cut-outs) [21,22]. Studies addressing complex three-dimensional curved glassware remain scarce. Unlike flat plates, high-velocity airflow over highly curved geometries intrinsically induces severe fluid distortions, such as flow separation, boundary layer detachment, and vortices. This results in highly non-uniform heat transfer coefficients at the bottom, rim, and diameter-transition areas.

Furthermore, the necessity of investigating this fluid distortion is heavily dictated by the specific material properties of the glassware. Unlike conventional soda-lime-silica glass, low-borosilicate glass possesses a significantly lower coefficient of thermal expansion and lower thermal conductivity. While these properties provide excellent intrinsic thermal stability, they make the physical tempering process notoriously difficult. Generating sufficient residual compressive stress in low-borosilicate glass demands extremely high cooling rates and intense aerodynamic quenching. Under such extreme conditions, any local non-uniformity in the convective heat transfer—exacerbated by the curved geometry—is severely amplified, making the product highly susceptible to fatal stress concentrations.

To address this gap, this study abandons the conventional uniform heat transfer assumption and establishes a high-fidelity fluid–thermal–structural sequential coupling model for low-borosilicate curved glassware using STAR-CCM+. By mapping the highly transient, 3D non-uniform aerodynamic boundaries directly onto the solid structural field, this research aims to quantify the exact modulation mechanism of geometric curvature on flow field distortion, heat transfer unevenness, and the ultimate residual stress distribution. Validated by polariscope experiments, this work provides a reliable quantitative foundation for industrial air-grid design and tempering optimization.

## 2. Experimental Method and Numerical Analysis

### 2.1. Experimental Method

**Figure 1:**
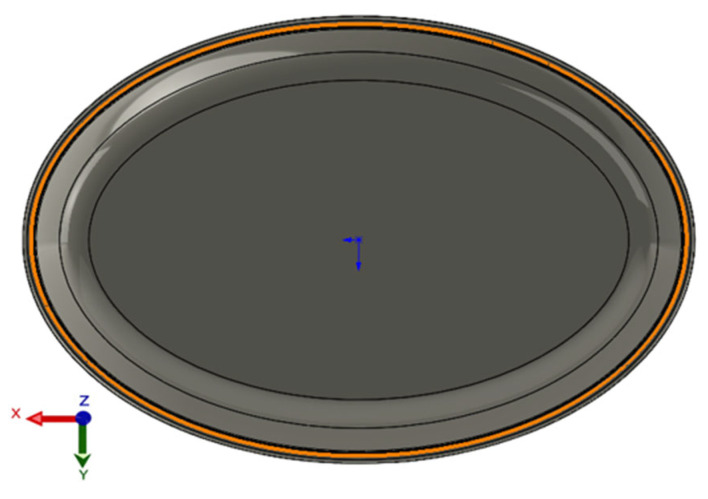
Schematic diagram of the glassware model.

**Figure 2:**
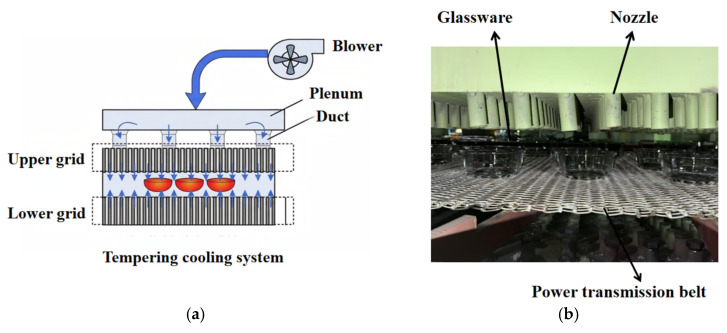
Experimental apparatus for the tempering and cooling process. (**a**) Schematic diagram and (**b**) experimental photograph of the tempering cooling device.

The glassware investigated in this study is a domestic oval glass baking pan. The geometric model of the baking pan was established in SolidWorks 2025 software, as illustrated in Figure 1. The dimensions at the rim are: length L = 400 mm, width B = 274 mm, height H = 75 mm, and thickness D = 6 mm. The maximum and minimum wall curvatures are 0.0535 and 0.0034, respectively. Figure 2 illustrates the schematic diagram and physical photograph of the glassware tempering cooling system. The system primarily comprises a blower, a plenum chamber, air ducts, an upper air grid, and a lower air grid. The upper and lower air grids are arranged symmetrically, featuring an array of circular nozzles with a diameter of 12 mm and a pitch of 60 mm. During the tempering and cooling process, the glassware, preheated to 650 °C, is conveyed underneath the air-grid nozzles via a mesh belt. High-velocity cold air jets are simultaneously and vertically impinged onto the glassware surfaces from both the upper and lower air grids. This causes the vessel surfaces to rapidly cool and contract, thereby inducing a temperature gradient along the thickness direction. Consequently, residual stresses characterized by internal tension and surface compression are developed, achieving the objective of strength enhancement.

It is worth noting that a sequential (one-way) fluid–thermal–structure coupling approach was adopted in this study rather than a fully two-way coupled formulation. Physically, the macroscopic deformation of the low-borosilicate glassware induced by thermal stress during the air cooling process is extremely small (typically on the order of micrometers). Such minimal geometric deformation does not exert any reverse interference on the surrounding high-speed aerodynamic flow field or the local convective heat transfer coefficients. Therefore, the sequential coupling strategy can guarantee sufficiently high predictive accuracy for the transient conjugate heat transfer and residual stress distribution, while maintaining numerical stability and significantly reducing the computational cost.

### 2.2. Fluid–Thermal–Structure Coupling Mechanism

**Figure 3:**
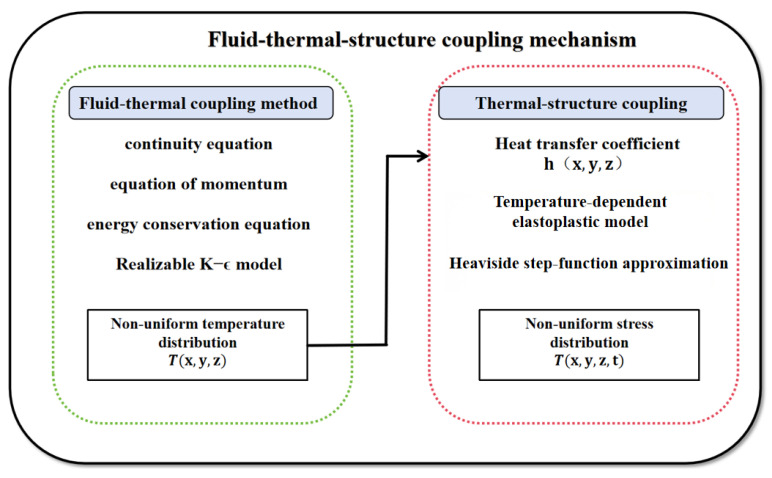
Flowchart of the sequential fluid–thermal–structural coupling simulation.

In this study, a fluid–thermal–structural coupling approach was employed to analyze the heat transfer and stress distribution characteristics of glassware during the air-cooling process. Figure 3 illustrates the flowchart of the sequential fluid–structure interaction (FSI) simulation. Initially, the finite volume method (FVM) is utilized to calculate the transient temperature field of the glass. Subsequently, using the obtained temperature results as predefined conditions, the finite element method (FEM) is applied to conduct the stress field simulation. Both the FVM and FEM computations are executed entirely within the simulation software STAR-CCM+ 2302.

The sequential fluid–thermal–structural coupling approach is physically grounded in the specific tempering conditions of this low-borosilicate glassware. Two fundamental physical characteristics justify this strategy:

(1) The thermal deformation of the glass during the rapid air-cooling process is constrained to the micrometer scale. Such minimal geometric variation does not exert detectable reverse interference on the high-speed aerodynamic flow field or the convective heat transfer coefficients.
(2) In the context of violent forced-convection quenching, the internal heat generated by phenomenological approximation of structural relaxation is infinitesimal compared to the massive external heat flux driven by the high-speed air jets. Consequently, the structural-to-thermal feedback loop is physically negligible, allowing for a decoupled simulation without loss of macroscopic predictive accuracy.

### 2.3. Establishment of Simulation Model

#### 2.3.1. Geometric Modeling

**Figure 4:**
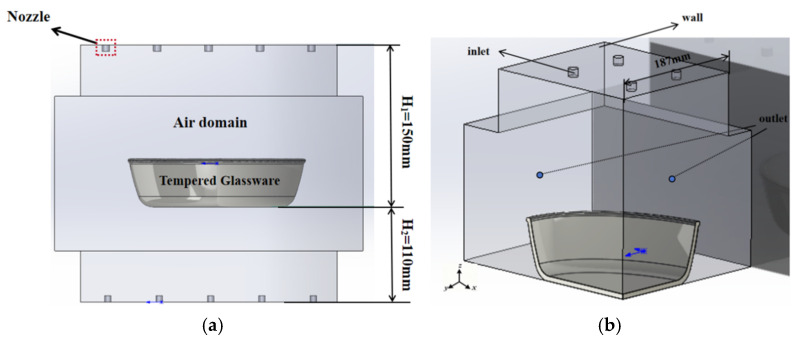
Geometric definition of the computational domain: (**a**) Computational domain; (**b**) 1/8th symmetry computational domain.

The geometric model of the glassware and its surrounding air domain was established using SolidWorks software, as shown in Figure 4. In Figure 4a, ambient-temperature air vertically enters the air domain through the air-grid nozzles and impinges upon the surface of the high-temperature glassware. The distances from the upper and lower air-grid nozzles to the bottom of the glassware are 150 mm and 110 mm, respectively. To capture the complete cooling process of the glassware, a full-scale model is adopted for the numerical simulation. Figure 4b illustrates the distribution of various regions within the 1/8 air domain model.

**Figure 5:**
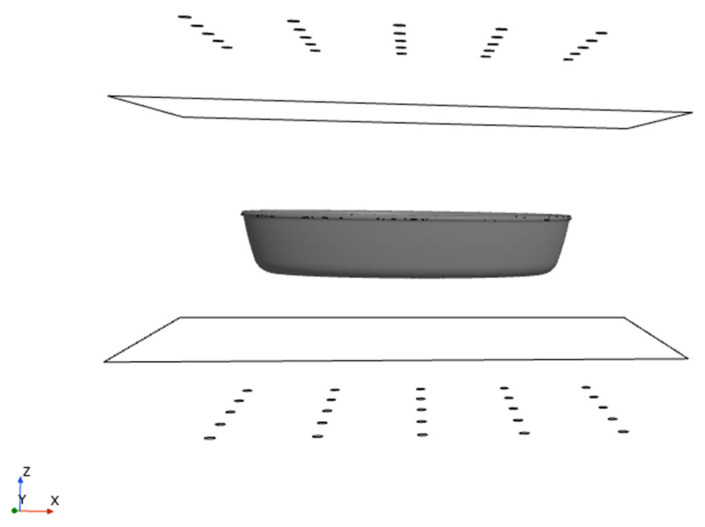
Fluid domain of the upper and lower wind grilles.

The aforementioned model was imported into the multi-physics simulation software STAR-CCM+, and boundary conditions were assigned based on the spatial distribution of different regions, as illustrated in Figure 5. Specifically, the uniformly arranged circular areas at the top and bottom serve as the air inlet nozzles, the central part corresponds to the glassware, and the lateral sides are designated as pressure outlets.

**Figure 6:**
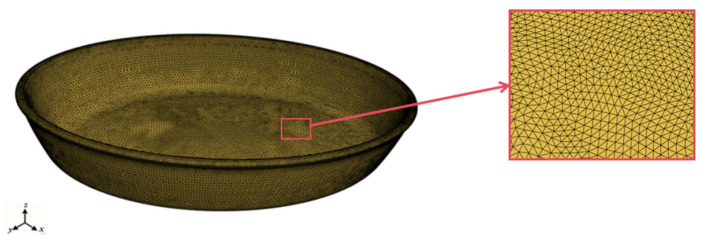
Glassware grid model.

**Figure 7:**
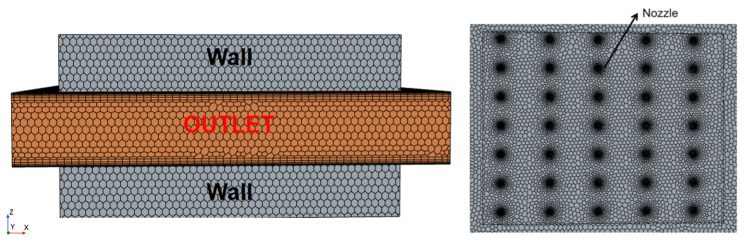
Air domain grid model.

**Figure 8:**
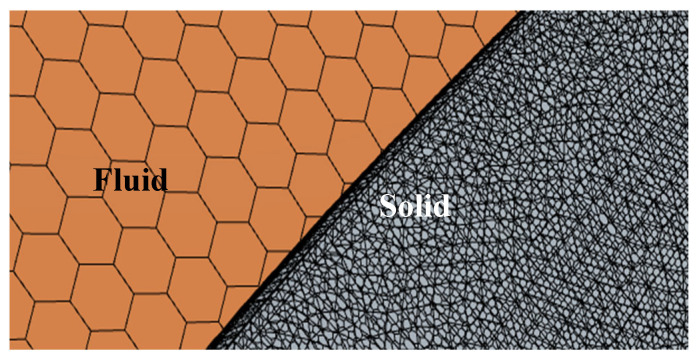
Interface mesh model.

Glass exhibits low thermal conductivity; during the rapid quenching phase, a significant temperature gradient is highly prone to developing along its internal thickness direction, thereby inducing thermal stresses, which constitutes the core mechanism of physical tempering. To balance computational accuracy and convergence for the complex curved features of glassware, the directed meshing technique is employed for grid generation. Specifically, the glassware is discretized using uniform tetrahedral thin-body meshes with a size of 2 mm, while the air domain utilizes uniform polyhedral meshes with prism layers at a size of 10 mm, as illustrated in Figure 6 and Figure 7, respectively. However, since a Boolean subtraction operation is required within the air domain to account for the glass geometry, mesh refinement was performed on the interface region between the air and the glass, as shown in Figure 8, where the minimum mesh size is 1mm.

#### 2.3.2. Material Properties and Boundary Conditions

**Figure 9:**
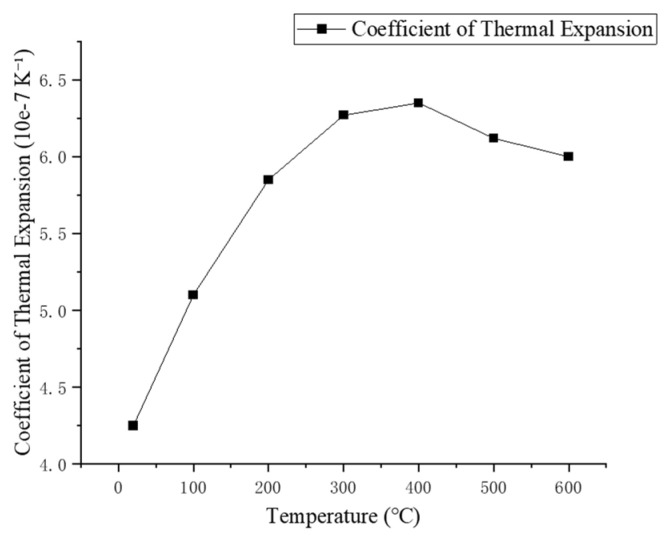
Thermal expansion coefficient–temperature curve of glass [16].

During the physical tempering process, glassware undergoes a drastic phase transition from a viscous liquid state to an elastic solid state. To capture the macroscopic variations of thermophysical properties across the glass transition temperature (Tg) without incurring the prohibitive computational costs of solving a full kinetic structural relaxation model (e.g., the TNM model or calculating dynamic fictive temperature), this study employs a phenomenological approach. The thermal conductivity (λ), specific heat capacity (C_P_), and coefficient of thermal expansion (γ) are defined as highly nonlinear [23], temperature-dependent piecewise functions or empirical curves. This temperature-dependent implementation effectively approximates the distinct thermophysical behaviors of the glass in both the liquid and solid states. The transitions of thermal conductivity and specific heat capacity are presented in Table 1, while the variation of the coefficient of thermal expansion with temperature is illustrated in Figure 9.

**Figure 10:**
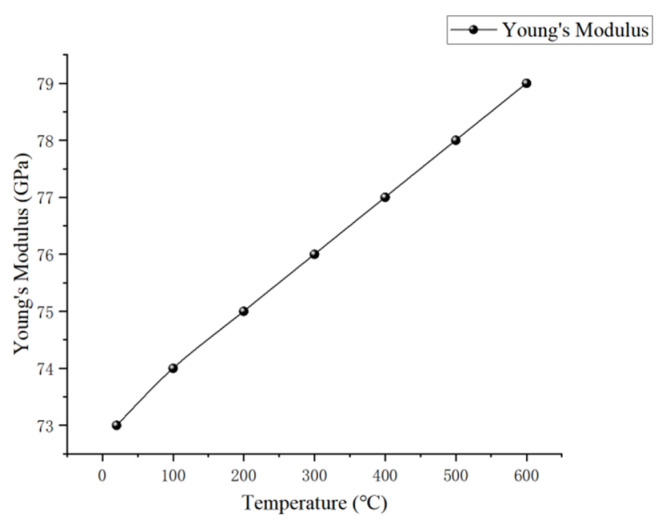
Variation of Young’s modulus with temperature [16].

As the temperature drastically decreases during the quenching process, the macroscopic viscosity of the glass climbs exponentially, and the microscopic chain segment mobility within the material is rapidly hindered. This severely suppresses and ultimately freezes the stress relaxation process in its mechanical response. Governed by the time-temperature superposition (TTS) principle, the material abruptly transitions from a fully relaxed viscous state to a rigid solid state. Once completely within the solid-state regime, dominated by the distinct “silica anomaly” characteristic of low-borosilicate glass, its Young’s modulus (E) exhibits a gradual increase with rising temperature due to the attenuation of thermal vibrational degrees of freedom, as illustrated in Figure 10.

In the structural field, to model the drastic stress relaxation near the glass transition region without incurring the prohibitive computational costs of full viscoelastic integration, a temperature-dependent elastoplastic constitutive model was implemented. The structural relaxation effect was phenomenologically approximated by a Heaviside step-function of yield stress across the glass transition temperature (Tg = 793.15 K) [24]. expressed as follows:

(1) $$\sigma_{Y}(T)=\left\{\begin{array}{c} 1.0\;\mathrm{Pa},\;\;T\geq 793.15\;K\\ 1.0\times 10^{10}\mathrm{Pa},\;\;T<793.15\;K \end{array}\right.$$

When the glass temperature exceeds, the yield stress is assigned a nominal value of 1.0 Pa, allowing thermal stresses to be instantaneously relaxed via plastic yielding, effectively mimicking the viscous liquid state. Conversely, when the temperature drops below, the yield stress abruptly increases to 10 GPa, forcing the material into a purely elastic regime. Within this solid-state regime, the material exhibits the distinct “silica anomaly” characteristic of low-borosilicate glass, where its Young’s modulus increases with rising temperature (as illustrated in Figure 10). This Heaviside approximation ensures high macroscopic predictive accuracy of residual stresses while maintaining solver stability during the sequential coupling process.

Furthermore, during the jet impingement tempering process, the cooling air is modeled as an incompressible ideal gas. Because the cold air (approximately 20 °C) ejected from the nozzles is instantaneously heated upon contacting the glass surface at temperatures up to 650 °C, its fluid dynamic properties undergo drastic variations. To accurately capture the distortion of the airflow boundary layer under the large temperature gradient in the near-wall region, the density ($\rho_{\mathrm{air}}$) of the fluid medium strictly adheres to the ideal gas equation of state:

(2) $$\rho_{\mathrm{Air}}=\frac{P}{R_{\mathrm{specific}}T}$$

where P is the absolute pressure, $R_{\mathrm{specific}}$ is the specific gas constant of air, and T is the local absolute temperature.

More importantly, the dynamic viscosity ($\mu_{\mathrm{Air}}$) and thermal conductivity ($\lambda_{\mathrm{Air}}$) of air increase significantly with increasing temperature. Within the STAR-CCM+ solver, this model employs Sutherland’s law—derived from the kinetic theory of molecular momentum exchange—to account for these temperature-dependent variations. Taking dynamic viscosity as an example, its correction function is expressed as follows:

(3) $$M_{\mathrm{Air}}=\mu_{\mathrm{ref}}{\left(\frac{T}{T_{\mathrm{ref}}}\right)}^{3/2}\frac{T_{\mathrm{ref}}+S}{T+S}$$

where $\mu_{\mathrm{ref}}$ is the reference dynamic viscosity of air at the reference temperature, and *S* is the Sutherland constant (typically *S* ≈ 110.4 K for air). The thermal conductivity $\lambda_{\mathrm{Air}}$ is also calculated using a correction law of the same form to account for temperature dependence.

As a diatomic gas mixture, when air is subjected to temperature differentials spanning hundreds of degrees Celsius, its internal molecular vibrational degrees of freedom are progressively excited with the rising temperature. This leads to a significant enhancement in its heat absorption capacity. Consequently, the specific heat capacity at constant pressure (${C_{P}}_{\mathrm{Air}}$) of the cooling airflow can no longer be treated as a constant. To accurately describe this property, a polynomial fitting function based on the absolute temperature (*T*) is implemented within the STAR-CCM+ solver in this study:

(4) $${C_{P}}_{\mathrm{Air}}(T)\;=\;a_{0}\;+{\;a}_{1}T\;+{\;a}_{2}T^{2}\;+\;a_{3}T^{3}\;+{\;a}_{4}T^{4}$$

where $a_{0}$ to $a_{4}$ are the polynomial coefficients for the specific heat capacity of air at standard atmospheric pressure.

**Figure 11:**
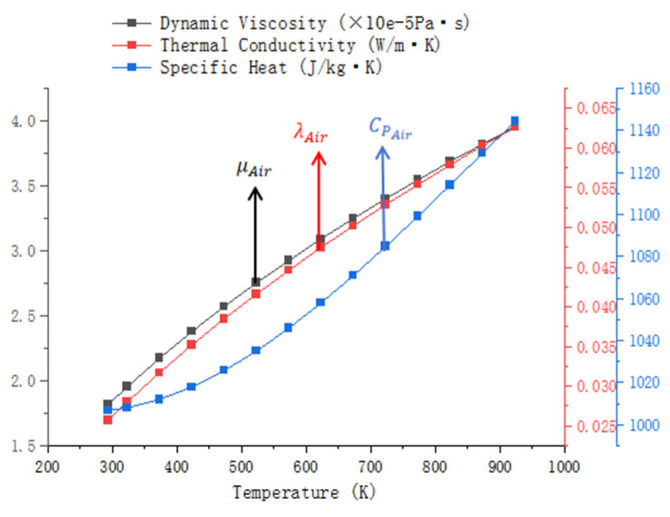
Variations of thermophysical properties of air with temperature.

Consequently, the temperature-dependent properties of air are plotted in Figure 11:

Regarding the computational domain, the boundary conditions were specified as follows:

(1) The nozzle array was designated as the inlet of the fluid domain with an applied velocity of 30 m/s, a temperature of 20 °C, and a turbulence intensity of 5%.
(2) The lateral boundaries of the air domain were defined as pressure outlets, with the pressure set to ambient atmospheric pressure and the temperature set to an ambient 20 °C.
(3) The glassware was defined as the fluid–solid interface and treated as a no-slip wall, with its initial temperature set to 650 °C.

During each coupling time step, STAR-CCM+ extracted the local transient convective heat transfer coefficient and the reference airflow temperature at the interface. Through spatial mapping techniques, these high-fidelity aerothermal boundaries were directly mapped onto the solid mesh surface of the glassware, thereby achieving a precise closed-loop calculation for the fluid, thermal, and structural fields.

It should be noted that while the experimental measurements indicate an inlet velocity fluctuation range of 28–32 m/s, a fixed mean velocity of 30 m/s was adopted for the boundaries in this simulation. This simplification is theoretically justified by the empirical correlation for forced convective heat transfer in turbulent jet impingement (Nu ∝ Re^m^, where m is typically between 0.5 and 0.8). Since the convective heat transfer coefficient (h) scales with the inlet velocity(v_in_) as h ∝ v_in_^m^, a measured velocity fluctuation of ±6.67% results in a maximum theoretical h variation of approximately ±5.3%(taking the conservative upper bound m = 0.8). Supplementary numerical tests at 28 m/s and 32 m/s confirmed that this thermal variation causes only a ±4.2% fluctuation in the absolute residual stress, while the fundamental spatial non-uniformity trends remain topologically consistent. Therefore, adopting 30 m/s as the representative stable boundary condition is scientifically robust for capturing the macroscopic stress evolution without loss of engineering accuracy.

While thermal radiation is present at the initial 650 °C, its impact was quantitatively assessed to evaluate the necessity of its integration. The equivalent radiative heat transfer coefficient (h_rad_) can be estimated using the following expression:

(5) $$h_{\mathrm{rad}}\;=\;\epsilon \cdot \sigma (T_{\mathrm{glass}}^{2}\;+\;T_{\mathrm{air}}^{2})(T_{\mathrm{glass}}\;+\;T_{\mathrm{air}})$$

where *ϵ* represents the surface emissivity of the glass, *T_glass_* is the glass surface temperature, and *T_air_* is the ambient cooling air temperature. Quantitatively, the calculated maximum equivalent radiative coefficient *h_rad_* ≈ 51.7 W/m^2^·K) accounts for merely 6–15% of the total surface heat flux compared to the intense forced convection (*h_conv_* = 300~800 W/m^2^·K). Furthermore, as the glass surface temperature drops precipitously within the initial seconds of quenching, this minor radiative contribution diminishes rapidly. Given that forced convection is the overwhelmingly dominant boundary mechanism, the radiative effect is treated as a secondary influence and is omitted to prioritize computational efficiency.

#### 2.3.3. Stress Analysis and Experimental Verification

**Figure 12:**
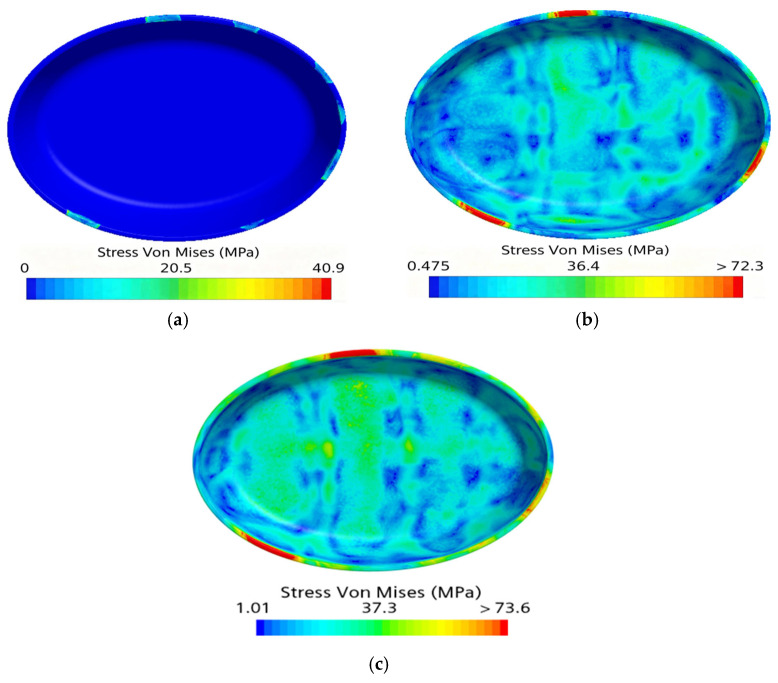
Stress distribution contours: (**a**) 10 s; (**b**) 120 s; (**c**) 260 s.

Figure 12 illustrates the stress simulation contour plots of the glassware under a 2 mm mesh at 10 s, 120 s, and 260 s. As indicated by the stress contours, at the rim of the glassware adjacent to the air grid, the maximum local stress can reach 73.6 MPa, whereas the overall stress level is maintained at approximately 40 MPa. This is in excellent agreement with the actual operating conditions and experimental measurements. Meanwhile, the inner surface region directly facing the air grid exhibits a relatively uniform stress distribution, generally remaining around 37 MPa.

**Figure 13:**
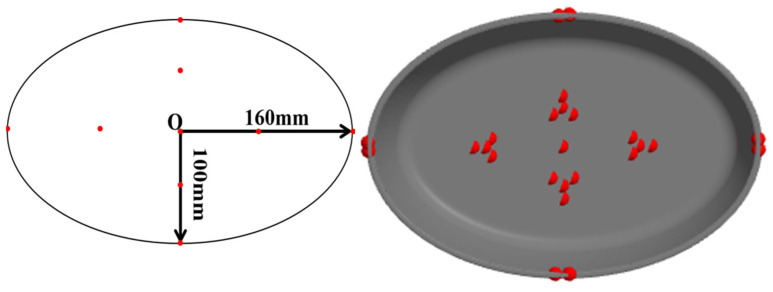
Schematic of stress detection locations.

**Figure 14:**
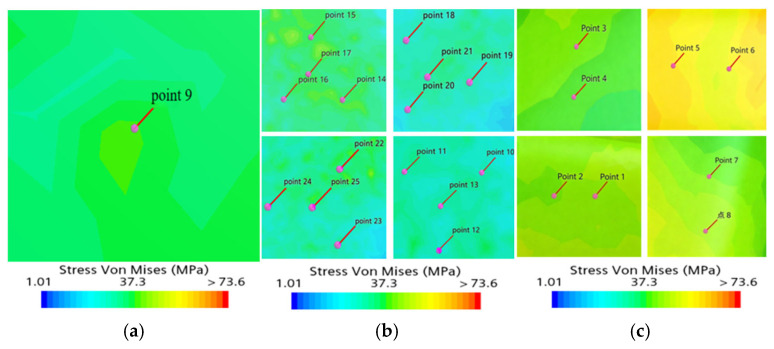
Stress simulation contour plots for different regions: (**a**) center point region; (**b**) inner surface region; (**c**) rim region.

To validate the accuracy of the established simulation model, the stress data obtained from the finite element analysis for various regions of the glassware were compared with the experimental measurements. The locations for stress detection are illustrated in Figure 13. In this model, a total of 25 observation points were selected across three distinct regions to extract the stress values, as depicted in Figure 13. Specifically, the first measurement location is at the center point of the glassware bottom. Subsequently, based on a proportional half-scale reduction of the bottom ellipse, points are selected at a radius of 12 mm at 120° intervals along the circumferential direction; the average of these four points is calculated as the representative stress value for this region. The third measurement region involves extracting 4 points uniformly along the circumferential direction at the rim of the glassware to compute the average value. Figure 14a–c present the stress simulation contour plots for the three regions: the bottom center point, the inner surface, and the rim of the glassware, respectively. As can be observed from the figures, the stress is the lowest at the bottom center point, followed by the inner surface, while the region with the maximum stress is located at the rim.

**Figure 15:**
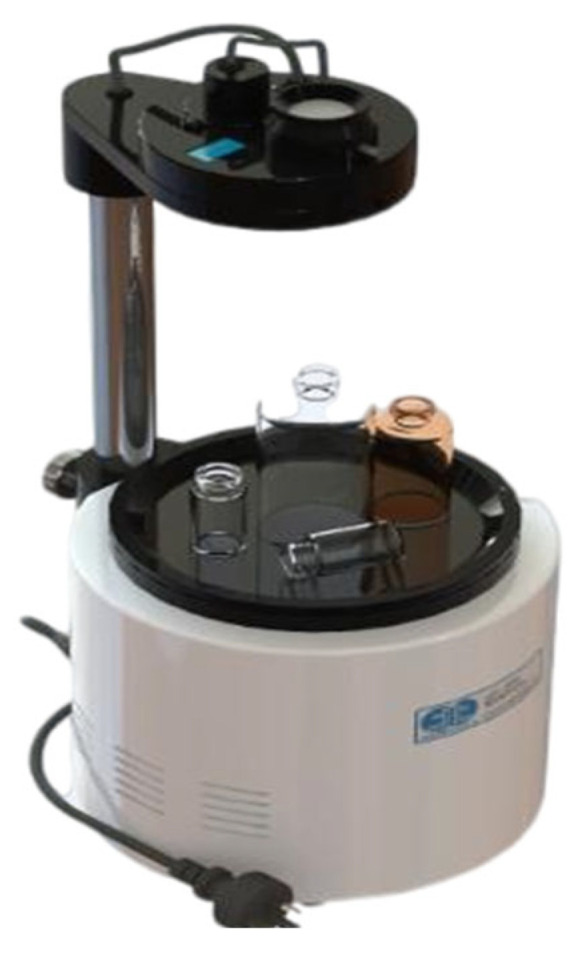
LZY-150D digital polariscope.

**Figure 16:**
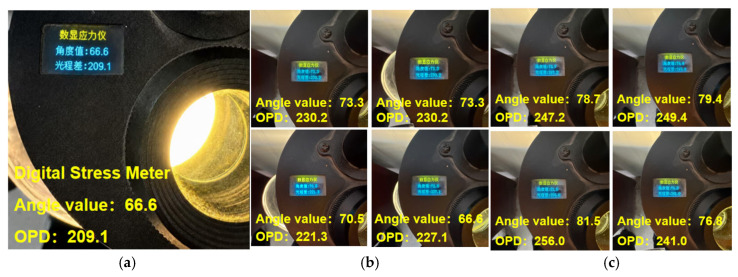
Experimental measurement results for different regions: (**a**) center point region; (**b**) inner surface region; (**c**) rim region, OPD is the abbreviation of optical path difference.

In the experimental phase, an LZY-150D digital polariscope (Figure 15) was employed to measure the stress at the designated locations of the glassware after the cooling process. The deflection angle or optical path difference (OPD) values at the corresponding measurement points were directly recorded from the instrument’s dual-numerical LED display, as depicted in Figure 16.

**Figure 17:**
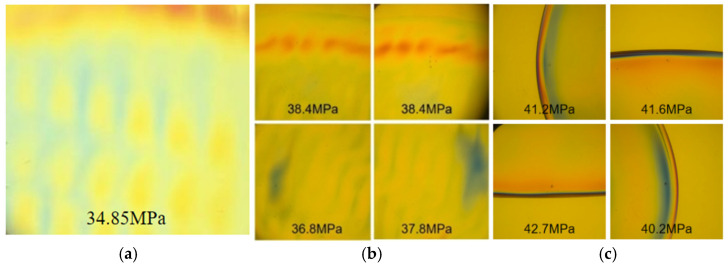
Images of measured locations and corresponding calculated stress values: (**a**) center point; (**b**) inner surface region; (**c**) rim region.

After directly acquiring the deflection angles or optical path difference (OPD) values for each region from the LED display, they were substituted into Equation (4) to calculate the corresponding stress values. The images of the measured locations along with their calculated stress values are presented in Figure 17.

(6) δ=T/t=θ×3.14/t

where δ is the stress of the sample (nm/mm); *T* is the optical path difference at the measured location of the sample (nm); t is the total thickness of the sample along the optical path at the measured location (mm); θ is the rotation angle of the analyzer (when the maximum stress is measured); 3.14 is the instrument constant when utilizing a white light source (with an effective wavelength of approximately 565 nm), indicating that a 1° rotation of the analyzer corresponds to an optical path difference of approximately 3.14 nm.

To rigorously validate the numerical model and quantify measurement uncertainty, the residual compressive stresses at three critical locations (center, bottom, and rim) were measured across five independent replicate samples using a digital polariscope. As shown in Table 2, the experimentally measured residual stress exhibits a spatially increasing trend from the center (35.35 MPa) to the rim (41.56 MPa).

**Figure 18:**
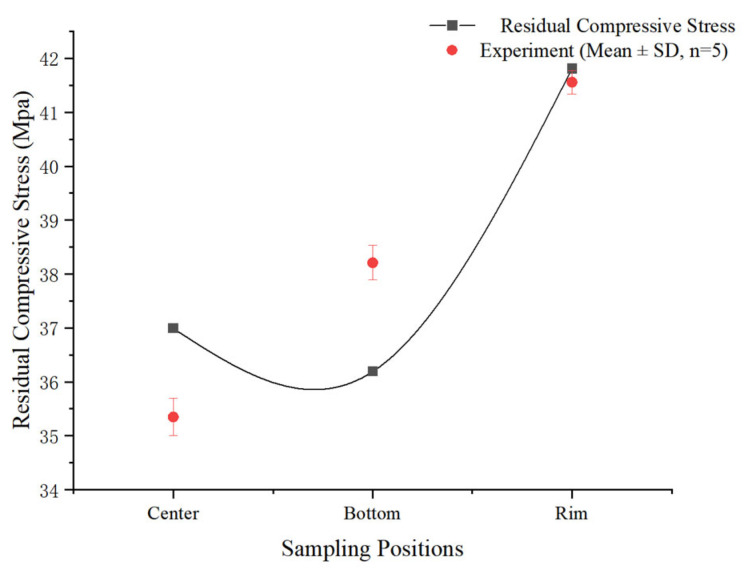
Comparison between simulated and experimentally measured residual compressive stresses at different locations of the glassware. Experimental data are presented as the mean ± standard deviation (n = 5).

The comparison between the simulated results and the experimental data is illustrated in Figure 18. The experimental measurements (red circles) exhibit excellent repeatability. The standard deviations range from ±0.22 to ±0.35 MPa, and the coefficients of variation (CV) are all below 1.0%, demonstrating the high stability of both the manufacturing and measurement processes. The simulated values (black squares) show high quantitative agreement with the experimental means. As summarized in Table 3, the relative errors of the stresses in all regions are well controlled within 6%. It should be acknowledged that, ideally, in-situ transient temperature and flow field measurements should be conducted to directly validate the CFD and thermal models. However, the glassware is quenched in a highly enclosed environment surrounded by dense high-pressure nozzle arrays. Installing intrusive sensors on the glassware would severely disrupt the original aerodynamic flow field, while non-contact infrared thermography is physically blocked by the equipment enclosure. Nevertheless, from the perspective of solid mechanics, the final residual stress generation is the direct macroscopic integral consequence of the entire transient thermal gradient history.

More importantly, the sequential fluid–thermal–structural coupling model accurately captures the spatially increasing trend of the residual compressive stress from the center toward the rim. This demonstrates that the model successfully reproduces the non-uniform heat transfer and varying cooling rates induced by the geometric curvature of the baking pan. Furthermore, although the digital polariscope is inherently limited to surface or near-surface stress measurements, the accurate prediction of the surface compressive stress provides robust macroscopic validation for the through-thickness stress distribution. According to the fundamental principles of solid mechanics, the residual stress distribution across the glass thickness must satisfy static equilibrium, meaning the surface compressive stresses and the core tensile stresses must balance. Therefore, the accurate experimental verification of the surface compressive stresses provides a necessary macroscopic boundary constraint for the through-thickness stress evolution. It should be acknowledged that satisfying this integral equilibrium does not uniquely validate the precise non-linear morphology of the internal tensile gradients. The accuracy of these complex internal profiles ultimately relies on the high-fidelity resolution of the transient internal thermal gradients captured by our sequentially coupled model.

In this study, the mesh independence was systematically evaluated in two sequential stages: the upstream fluid domain and the structural glassware domain. Because the rapid quenching is a violently forced convection process, the aerodynamic velocity fundamentally dictates the thermal boundaries. Therefore, prior to the structural evaluation, the mesh independence of the fluid domain was verified first. Four different polyhedral mesh densities (10 mm, 5 mm, 2 mm, and 1 mm) were tested by monitoring the time-averaged local maximum velocity within the fluid domain (which locally accelerates beyond the 30 m/s inlet velocity due to geometric constriction and the rapid thermal expansion of the heated air). A sequential convergence analysis revealed excellent flow field stability: the relative deviations between the 10 mm and 5 mm meshes, and between the 5 mm and 2 mm meshes, were merely ~0.61% and ~1.04%, respectively. Further refining the mesh from 2 mm to 1 mm yielded an extremely negligible deviation of approximately 0.44%. Considering that the 1 mm mesh significantly increased the computational cost without a proportional gain in accuracy, the 2 mm fluid mesh was ultimately selected as the optimal configuration. This aerodynamic convergence provides a reliable foundation for the stability of the transient thermal fields.

**Figure 19:**
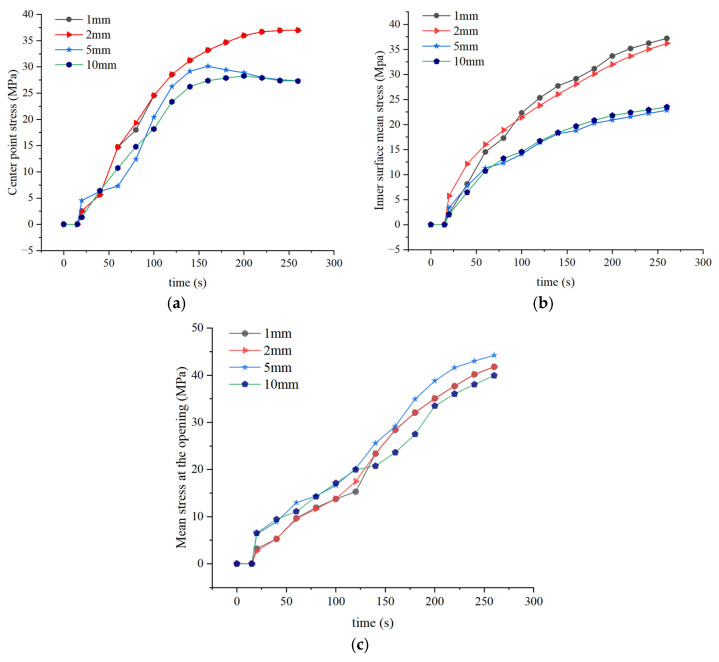
Stress–time variation curves for different regions: (**a**) center point stress; (**b**) inner surface average stress; (**c**) rim average stress.

Subsequently, based on these converged thermal loads mapped from the fluid domain, the structural mesh independence of the glassware was evaluated. Under the boundary conditions of V = 30 m/s, T = 293.15 K, and H1 = 150 mm, solid mesh sizes of 1 mm, 2 mm, 5 mm, and 10 mm were applied to the glassware, corresponding to element counts of 5,546,563, 2,205,300, 1,414,988, and 1,240,428, respectively. The structural mesh independence analysis was conducted for the center point, inner surface, and rim regions. Figure 19 presents the stress–time variation curves of the glassware across these different regions and mesh sizes.

As can be observed from Figure 19a,c, the stress variation trends of the 1 mm and 2 mm meshes are identical, with their stress values essentially overlapping, whereas the 5 mm and 10 mm meshes exhibit merely similar trends. In Figure 19b, a distinct difference in the stress variation trends is observed between the finer meshes (1 mm and 2 mm) and the coarser meshes (5 mm and 10 mm). To validate the accuracy of the meshes, the final simulated stress values were compared with the final experimental measurements, as presented in Table 4, Table 5 and Table 6. The relative errors for both the 1 mm and 2 mm meshes across different regions are all less than ±6.5%. Therefore, to ensure both the accuracy and computational efficiency of the simulation, the 2 mm mesh was adopted for this study.

## 3. Results and Discussions

### 3.1. Flow Field Characteristics

**Figure 20:**
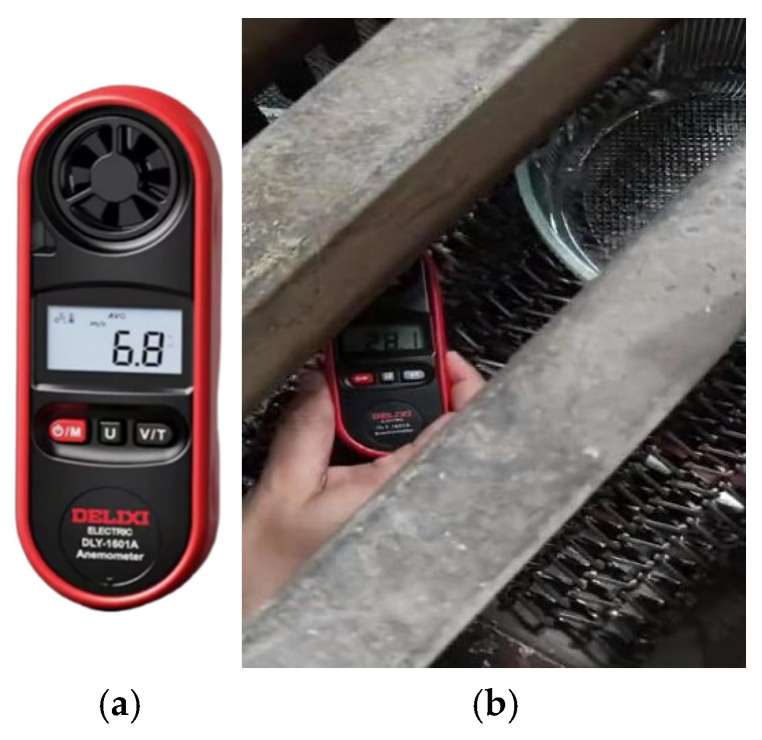
Experimental measurement apparatus and interface: (**a**) DLX-1601A anemometer; (**b**) actual temperature measurement display.

In the physical experimental measurements, a DLX-1601A anemometer (Figure 20) was utilized to measure the initial airflow velocity near the nozzle exit, yielding a velocity range of 28–32 m/s with an air temperature Tair ≈ 20 °C. Based on this experimental boundary, corresponding inlet parameters were specified in the numerical simulation. Specifically, the air density was dynamically calculated adhering strictly to the ideal gas law, and the Realizable K-ε turbulence model was selected to accurately capture the flow field dynamics characteristics under complex curved surfaces.

**Figure 21:**
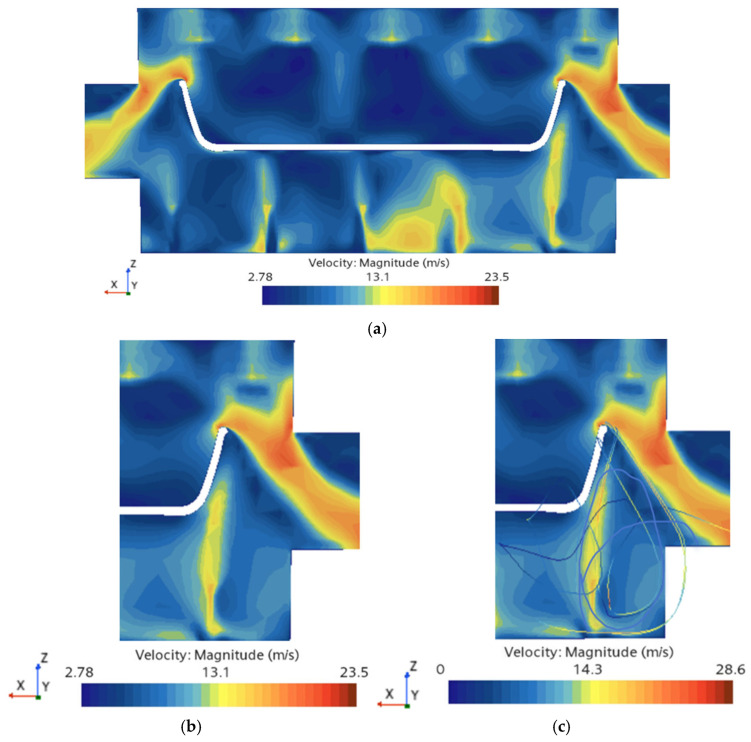
Flow field characteristics in the computational domain: (**a**) velocity magnitude distribution on the Y = 0 plane; (**b**) localized flow field distribution; (**c**) localized flow field streamlines.

To intuitively analyze the interaction laws between the cooling airflow and the glassware, the velocity magnitude distribution contour plot on the symmetry plane (Y = 0) of the computational domain at 100 s was extracted, as illustrated in Figure 21a. Regarding the local flow field around regions with abrupt geometric curvature changes, such as the rim of the glassware, Figure 21b details the characteristics of its velocity gradient distribution. As observed from the figures, when the vertically downward high-velocity airflow impacts and flows around the curved bottom corners of the glassware, it is highly prone to inducing flow separation and vortex generation. This local aerodynamic distortion significantly alters the velocity distribution of the surrounding flow field, generating a localized acceleration effect. To visualize the velocity trajectories more intuitively, streamlines were generated to observe the variation of air velocity near the vortices, as depicted in Figure 21c. The maximum velocity can reach 26 m/s at the curved section of the glassware, while it reaches 28.6 m/s at the exit boundary.

While the existence of flow separation and vortices is a classical aerodynamic phenomenon over curved surfaces, its physical impact on the specific tempering process of low-borosilicate glass is profound and must be quantified. Quantitatively, this curvature-induced flow separation drastically deteriorates the local convective heat transfer efficiency. According to the extracted CFD data, in the direct jet impingement region (the stagnation zone at the bottom), the local convective heat transfer coefficient (h_conv_) reaches peak values ranging from approximately 300 to 360 W/(m^2^·K). Conversely, in the severe vortex and flow separation regions near the curved rim, the local airflow stagnates or recirculates, causing the h_conv_ to drop precipitously to roughly 140–160 W/(m^2^·K). This massive spatial variation—a nearly 50% to 60% reduction in local cooling intensity—represents the fundamental quantitative driver for the severe non-uniformity in the subsequent transient temperature and residual stress fields.

### 3.2. Heat Transfer Characteristics

**Figure 22:**
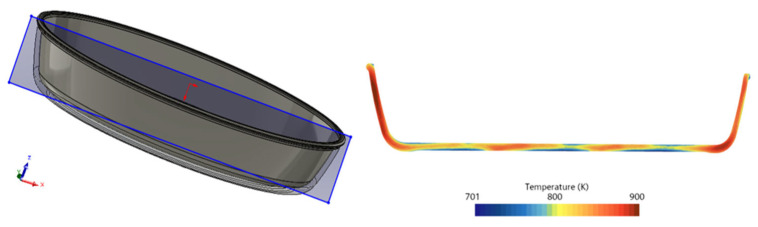
Cross-sectional model and temperature field characteristics: Cross-sectional model and temperature contour plot along the thickness direction.

To thoroughly investigate the thermal non-uniformity along the thickness direction of the glassware during the quenching process, a quantitative analysis was performed on the temperature evolution laws within the characteristic regions. Regarding the deployment of measurement points, temperature nodes at the inner surface, the center layer, and the outer surface were extracted along the wall thickness direction, using the stress-concentrated inner surface region as the benchmark. Figure 22 illustrates the cross-sectional model of the glassware and the transient temperature field distribution characteristics along the thickness direction after quenching for 10 s. By extracting the transient temperature history data of these characteristic regions and calculating their spatially averaged temperatures, the cooling kinetics characteristics of the three regions were further quantified and compared.

**Figure 23:**
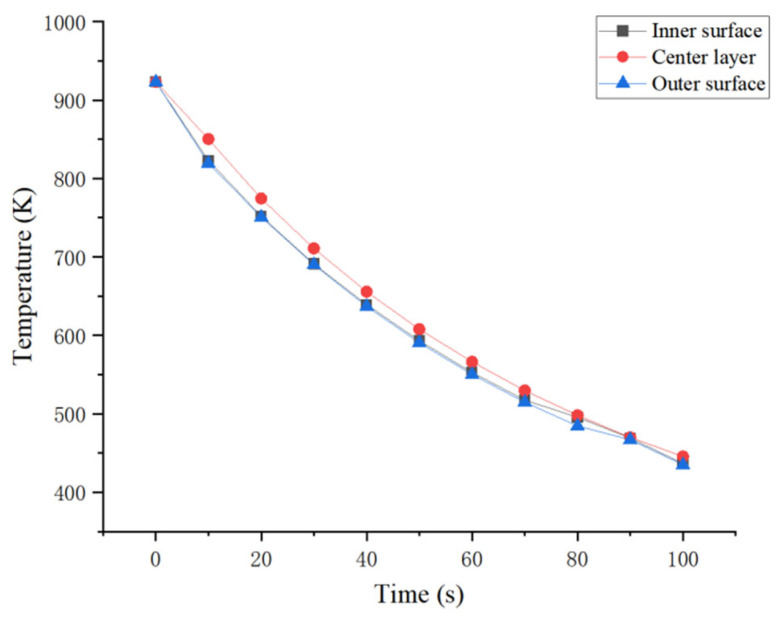
Temperature variation curves over time along the wall thickness direction.

The comparative results are illustrated in Figure 23. As observed from the figure, within the quenching cycle of 0–100 s, the overall temperature decay trends of both the inner and outer surfaces exhibit a high degree of consistency. Concurrently, the temperature of the center layer remains significantly higher than that of both surfaces throughout the entire cooling stage, establishing a pronounced temperature gradient along the wall thickness direction of the glassware. Because the inner surface presents a semi-enclosed cavity geometric feature, the flow of the cooling airflow within the cavity is highly prone to inducing a stagnation effect. This layout mitigates the airflow turbulence and relatively weakens the surface heat transfer coefficient, thereby restricting the gas–solid convective heat transfer intensity in this region (with the local h_conv_ averaging only 200–250 W/(m^2^·K), which is significantly lower than the 300–360 W/(m^2^·K) achieved at the unconstrained outer bottom stagnation zone). In contrast, the external flow field surrounding the outer surface is more open, allowing for more sufficient cooling airflow and higher convective heat transfer efficiency. Regarding the center layer of the glassware, it cannot directly undergo convective heat transfer with the cooling medium; its heat dissipation relies entirely on thermal conduction from the interior of the glass matrix toward both surfaces. Owing to the inherently low thermal conductivity of the glass material, a larger internal thermal resistance is produced, resulting in the most sluggish cooling response.

**Figure 24:**
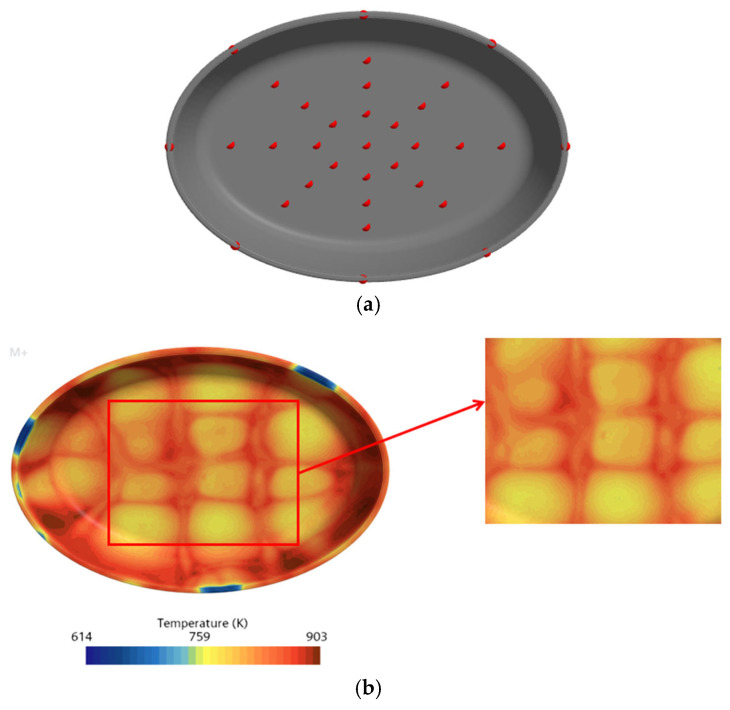
Surface sampling scheme and transient temperature distribution: (**a**) surface sampling point layout; (**b**) temperature distribution on the inner surface of the glassware at 10 s with a localized magnified view.

The uniformity of temperature distribution is of paramount importance to the quality of glass tempering. Given the profound influence of thermodynamic characteristics on the stress distribution of glass, a further in-depth analysis was conducted focusing on the temperature distribution uniformity of the glassware surface layer. To ensure sufficient sampling of the surface layer, measurement points were selected based on the geometric scaling of the elliptical profile of the bottom surface. On each ellipse, points were sampled at 45° angular intervals across three distinct layers, and the bottom region was sampled according to the inner surface point selection method, as illustrated in Figure 24a. To intuitively observe the surface temperature distribution, the temperature distribution of the glassware at 10 s, along with its localized magnified views, was captured, as depicted in Figure 24b.

As indicated by the figure above, during the air-cooling process, the cooling rate is significantly faster in the regions directly aligned with the air nozzles, and heat is transferred from the high-temperature zones to the low-temperature zones. Because the highly distorted aerodynamic field causes the local convective heat transfer coefficient (h_conv_) to vary drastically across the curved geometry—plummeting from approximately 360 W/(m^2^·K) at the stagnation zone to ~140 W/(m^2^·K) in the vortex regions—it fundamentally drives a severely uneven temperature distribution during heat transfer. This confirms that the severe thermal non-uniformity is primarily governed by the extreme spatial fluctuations in external aerodynamic convection, rather than the minor non-linear variations in the glass’s intrinsic thermal conductivity. Furthermore, the units at the rim possess three cooling surfaces, whereas the center units have only two, indicating that the temperature distribution is governed by multiple coupled factors. Therefore, this study first introduces the flow uniformity index F, which is constructed based on the statistical relative standard deviation (RSD) [23]. Concurrently, considering that the temperature field within the spatially discretized mesh domains in finite element analysis exhibits highly consistent data structure characteristics, a temperature uniformity index (U_T_) is defined in this study, using the statistical philosophy of flow uniformity as a cornerstone.

Physically, this dimensionless index represents the macroscopic spatial consistency of the transient temperature distribution, which directly governs the magnitude of subsequent thermal stresses. From a statistical perspective, UT is essentially the engineering counterpart of the classical Area-Weighted Coefficient of Variation (CV) or Relative Standard Deviation (RSD). While CV emphasizes data dispersion, UT is mathematically normalized to yield a consistency percentage, bridging rigorous statistical mechanics with intuitive industrial quality assessment. Furthermore, because it is dimensionless and normalized by the global mean temperature, UT is completely independent of specific geometric scales, allowing for universal transferability to evaluate other flat or complex curved structures. Regarding its sensitivity to sampling density, the evaluation of UT is performed via discrete integration over the surface mesh. Therefore, its numerical stability is strictly guaranteed as long as the computational domain satisfies the grid independence criteria, rendering it insensitive to localized mesh density fluctuations.

(7) $$U_{T}=\left\{1-{\left[\frac{1}{n_{t}}{\sum_{i=1}^{n}\left(\frac{T_{i}-\bar{T}}{\bar{T}}\right)}^{2}\right]}^{\frac{1}{2}}\right\}\;\times \;100\%$$

where $n_{t}$ is the total number of discrete sampling points or finite element mesh nodes from which temperature data are extracted; $T_{i}$ represents the thermodynamic temperature of the *i*-th node (K); and $\bar{T}$ denotes the arithmetic mean value of the corresponding variable within the entire sampling region.

**Figure 25:**
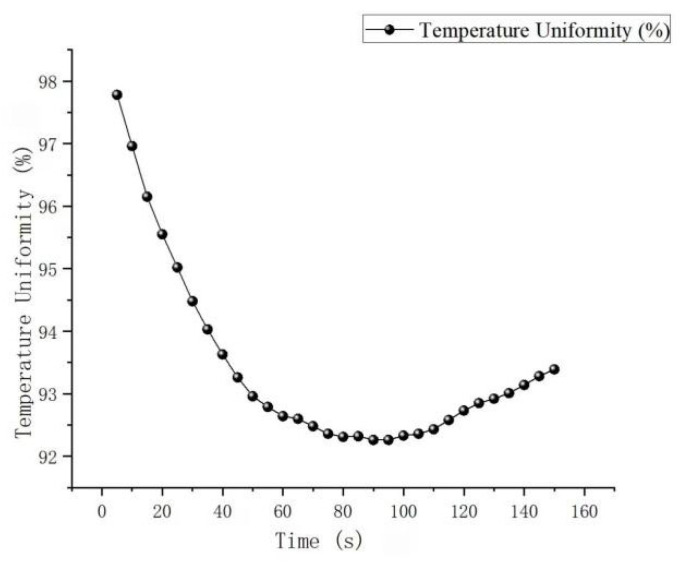
Temperature uniformity variation curves of the glassware surface layer over time.

The numerical simulation of the glass quenching process was conducted using STAR-CCM+ software, and the results are presented in Figure 25. Under the boundary conditions incorporating a variable heat transfer coefficient (VHTC), the temperature uniformity of the glassware surface layer does not exhibit a monotonic decay; instead, it demonstrates a distinctive and non-linear time-varying characteristic of “destruction and subsequent reconstruction.” In the initial cooling stage (0–90 s), the localized strong convection effects induced by the nozzle jet flows, combined with the geometric discrepancies of multiple cooling surfaces at regions such as the rim, trigger intense transient heat conduction. This phenomenon rapidly disrupts the original thermal equilibrium state of the glass, causing the surface temperature uniformity to drop sharply from an initial 97.8% to approximately 92.2%. Although a uniformity index of 92.2% (i.e., an ≈7.8% spatial deviation) might appear minor as a percentage, its absolute physical consequence is substantial. At elevated quenching temperatures (e.g., around 700 K), an 8% spatial deviation translates to an absolute surface temperature gradient of approximately 50~60 K. Given the high stiffness and brittleness of low-borosilicate glass, such a massive absolute temperature differential is sufficient to induce critical localized thermal stresses and trigger spontaneous breakage, underscoring the vital industrial significance of closely monitoring this index. However, during the mid-to-late cooling stage (90–150 s), as the overall temperature of the glass decreases, the intensity of the surface forced convective heat transfer attenuates. Concurrently, the internal thermal conduction effect, through which heat is transferred from high-temperature zones to low-temperature zones, gradually becomes dominant. This effectively flattens the localized surface temperature gradients, prompting the temperature uniformity to gradually recover to 93.4%.

### 3.3. Stress Field Characteristics

**Figure 26:**
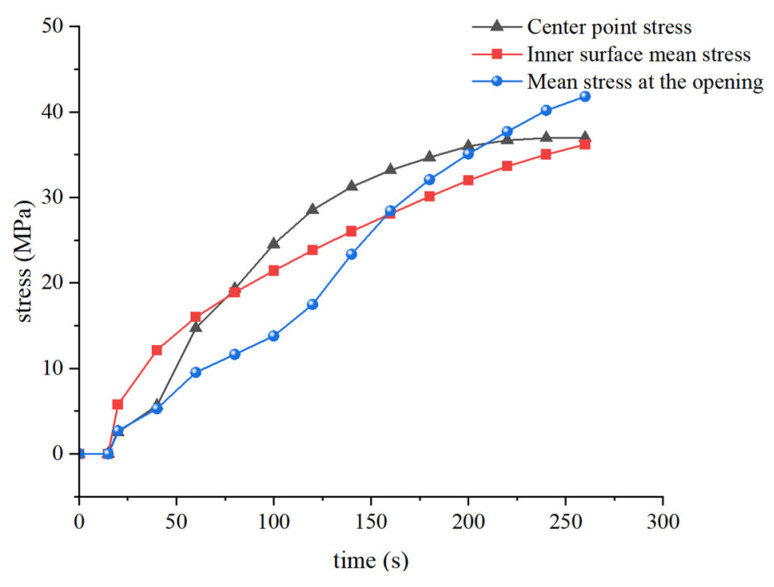
Stress–time variation curves in different regions.

As described above, during the air-cooling process, the dominant heat transfer mechanisms at the glass surface and within its interior are convective heat transfer and thermal conduction, respectively. This mechanistic discrepancy leads to varying cooling rates along the thickness direction of the glass, and the resulting non-uniform temperature gradients ultimately evolve into residual stresses. In this section, a stress uniformity analysis was performed based on the stress simulation results of different regions, and the stress curves of various regions over time are presented in Figure 26. During the high-temperature stage of glass quenching at 0–15 s, the material exhibits significant viscous flow characteristics. Physically, according to the Maxwell viscoelastic theory [25], the structural relaxation time of the system at this stage is extremely short, leading to the rapid dissipation of thermal stresses [26]. To phenomenologically mimic this instantaneous stress relaxation without directly solving the full viscoelastic constitutive equations, this study utilizes the aforementioned temperature-dependent thermo-elastoplastic approach. By assigning a near-zero nominal yield stress in this high-temperature regime, any thermally induced stresses are instantaneously accommodated via plastic yielding. Consequently, no macroscopic stress accumulation is observed, maintaining the stress level near 0 MPa.

Similar to the temperature uniformity index, the physical significance and general applicability of the stress uniformity index (U_S_) are also firmly grounded in classical statistics. Physically, U_S_ characterizes the spatial consistency of the residual compressive stress, which directly dictates the macroscopic structural integrity and the potential risk of localized weak points (origins of spontaneous breakage) in the tempered glass. Mathematically, U_S_ is also derived from the Area-Weighted Coefficient of Variation (CV). By converting the absolute spatial stress deviation into a dimensionless consistency percentage, U_S_ maintains universal transferability across different glass geometries. Additionally, because its calculation relies on the fully converged, grid-independent mesh nodes, the U_S_ metric inherits the same numerical robustness as U_T_, remaining highly insensitive to sampling density variations and providing a reliable macroscopic evaluation of the tempering quality, which can be expressed as follows:

(8) $$U_{S}\;=\;\left\{1-{\left[\frac{1}{n_{z}}{\sum_{i=1}^{n}\left(\frac{S_{i}-\bar{S}}{\bar{S}}\right)}^{2}\right]}^{\frac{1}{2}}\right\}\;\times \;100\%$$

where $n_{z}$ is the total number of discrete sampling points or finite element mesh nodes from which stress data are extracted; $S_{i}$ represents the residual stress of the *i*-th node (MPa); and $\bar{S}$ denotes the arithmetic mean value of the corresponding variable within the entire sampling region.

**Figure 27:**
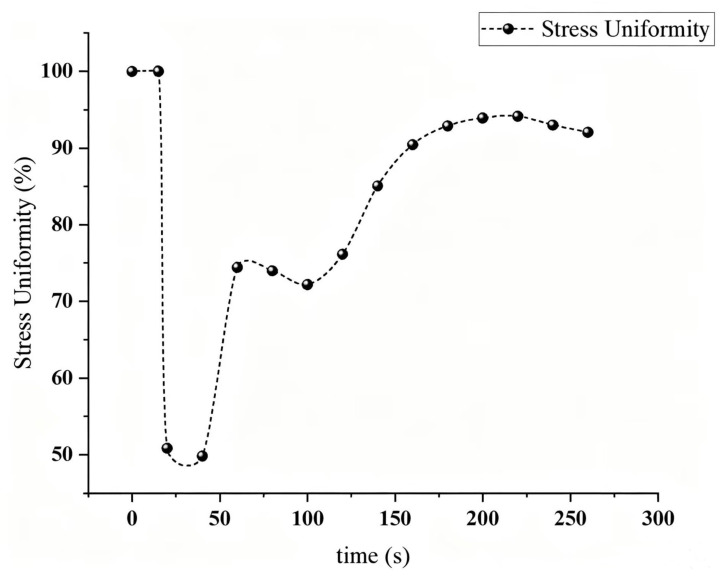
Stress uniformity curves of the glassware.

As illustrated in Figure 27, during the initial 15 s stage, the glass remains in a fully relaxed state, with the stress uniformity maintained at 100%. When the material cools below the glass transition temperature (Tg), stress accumulation initiates across various regions, causing the stress uniformity to drop sharply to its minimum point of 49.82%. Although U_S_ and U_T_ share the same normalized mathematical framework, their temporal evolutions reveal independent physical insights. While the minimum thermal uniformity (U_T_) merely dropped to 92.2%, the stress uniformity (U_S_) plummeted to 49.82%. This massive discrepancy demonstrates that during the phase transition, even minor thermal heterogeneities are drastically amplified into severe structural stress gradients, highlighting the unique necessity of the U_S_ metric. As cooling intensifies, the glass matrix progressively transitions into an elastic state, during which the residual stress values surge dramatically, resulting in a stress uniformity recovery to 74.44%. However, owing to the massive spatial gradients in local cooling rates (e.g., h_conv_ dropping from 360 to 140 W/(m^2^·K)), the volumetric shrinkage across different regions is highly asynchronous. This localized asynchronous phase transition induces the transient structural fluctuations, resulting in an oscillation amplitude of 3.95%. As the temperature gradient along the thickness direction gradually diminishes and the overall temperature falls thoroughly below the transition point, the heat exchange process stabilizes. The thermal perturbations previously induced by localized temperature differences are completely absorbed within the material interior. Consequently, the residual stress field is ultimately “locked-in” and tends toward an adaptive equilibrium, prompting the stress uniformity to rise again to 94% and eventually stabilize above 90%.

**Figure 28:**
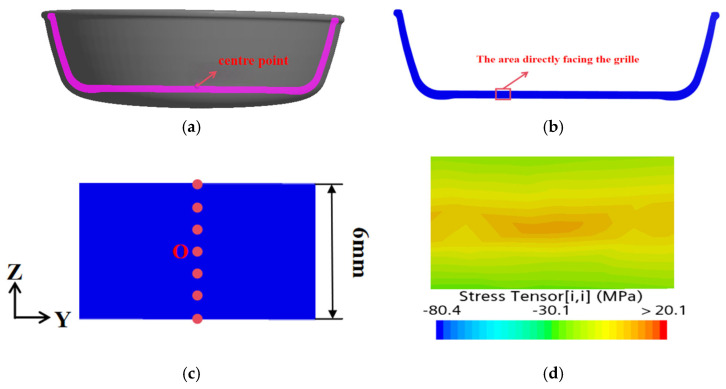
Schematics of the cross-sectional model and stress post-processing: (**a**) thickness cross-section intercepted through the center point; (**b**) selected region directly aligned with the air nozzles on the cross-section; (**c**) measurement point layout along the thickness direction within the cross-sectional region; (**d**) simulated residual stress contour plot of the section.

**Figure 29:**
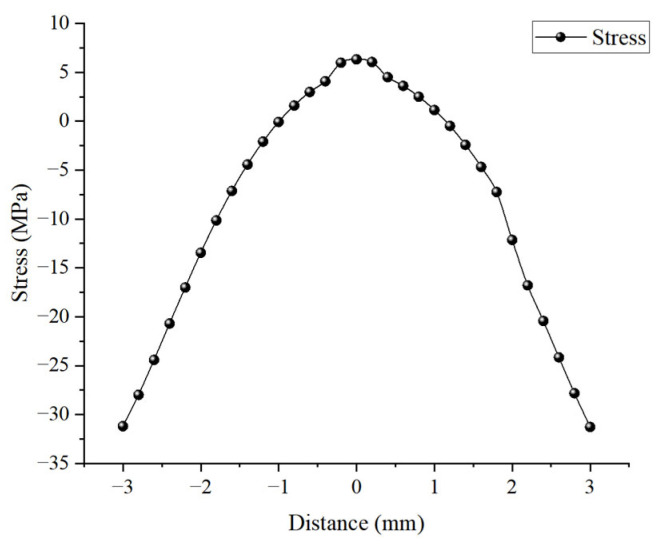
Stress distribution curves along the thickness direction.

To further investigate the stress distribution along the thickness direction, the plane located at the center point was intercepted along the Y-axis, and the regions directly aligned with the upper and lower air nozzles were selected. A line probe was established to measure the stress distribution along the thickness direction within this region, based on which the stress distribution curves were eventually plotted. Specifically, Figure 28a shows the thickness cross-section intercepted through the center point; Figure 28b indicates the selected region directly aligned with the air nozzles on the cross-section; Figure 28c displays the measurement point distribution along the thickness direction within this cross-sectional region; and Figure 28d illustrates the simulated stress distribution contour plot of this section. After arranging the probes in this region, the stress distribution curves along the thickness direction were plotted at every 0.2 mm of thickness, as shown in Figure 29. As observed from the curves, the stress profile along the thickness direction approximately exhibits a parabolic shape, wherein the surface compressive stress is approximately 31.1 MPa, and the internal tensile stress is around 5.7 MPa. Consequently, the residual stress value at the bottom of the glassware is approximately 36.8 MPa, which deviates from the previously mentioned experimentally measured stress value by only 1.41 MPa.

To quantitatively describe the parabolic profile of the through-thickness stress distribution under this specific quenching condition, a fourth-order empirical polynomial was fitted to the simulated data:

(9) $$S(x)\;=\;5.67748\;+\;0.78044x\;-\;5.01691x^{2\;}-\;0.09886x^{3\;}+\;0.09544x^{4}$$

where *x* is the position coordinate along the wall thickness direction of the glassware (in mm), and *S*(*x*) represents the local residual stress value (in MPa). It is crucial to emphasize that this polynomial is not a universal predictive model. As an empirical mathematical fit, it lacks a fundamental physical derivation and is strictly limited to the specific geometry and cooling conditions investigated in this study. It serves merely as a phenomenological descriptor to illustrate the parabolic nature of the stress gradient.

Compared to existing tempering studies that primarily focus on flat glass [18,19] or assume idealized uniform heat transfer coefficients [10,15], the curved glassware in this study exhibits distinct multiphysics characteristics. Unlike flat plates, where stress concentrates strictly near the edges, our results reveal a continuous, broad, spatially increasing trend of residual stress from the center (35.35 MPa) to the rim (41.56 MPa). Moreover, while the local through-thickness stress maintains the classic parabolic profile (surface compression balancing internal tension) characteristic of tempered glass, the amplitude of this parabola fluctuates significantly across the 3D geometry, in stark contrast to the spatially uniform profiles predicted in flat plate models. This discrepancy demonstrates that 3D geometric curvature fundamentally alters the aerodynamic flow, inducing macroscopic flow separation and extending the non-uniform stress transition zone. Furthermore, instead of the monotonically converging uniformity predicted by uniform-HTC models [10,15], our simulation captures a pronounced nonlinear “destruction and subsequent reconstruction” trend. While recent high-fidelity CFD studies on flat glass [22] noted similar fluctuations, the complex curvature of the baking pan severely exacerbates the initial uniformity drop (down to ~49.8% for stress) due to localized jet stagnation in the cavity and multi-surface cooling at the rim. This comparison underscores the critical necessity of integrating realistic fluid dynamics when predicting the tempering behavior of shaped glassware.

### 3.4. Comprehensive Discussion on Tempering Mechanisms

To synthesize the numerical observations above, it is essential to distinguish the apparent multiphysics correlations from the fundamental underlying mechanisms. The severe residual stress non-uniformity observed in this study is not merely correlated with the distorted aerodynamic field; it is mechanically driven by the thermal expansion mismatch during the asynchronous phase transition across the glass transition temperature (Tg). Specifically, the curvature-induced flow separation creates massive convective heat transfer gradients (varying from 360 to 140 W/(m^2^·K)). This causes the surface layer in the stagnation zone to cool rapidly below Tg and solidify into a rigid elastic state, while the regions enveloped in vortices (and the internal core) remain in a viscous state above Tg. When these hotter regions subsequently attempt to undergo volumetric shrinkage, they are mechanically constrained by the already solidified external network. This asynchronous thermal contraction and structural mismatch permanently lock in the massive spatial stress gradients.

Furthermore, this mechanism is uniquely amplified by the intrinsic material properties of low-borosilicate glass. Compared to conventional soda-lime glass, low-borosilicate glass possesses a significantly lower coefficient of thermal expansion (CTE) and poorer thermal conductivity. Achieving the required thermal expansion mismatch to generate sufficient compressive stress necessitates extreme quenching intensities. However, forcing high-velocity airflow over 3D curved surfaces inevitably provokes more violent flow separation and vortex shedding. Thus, the inherent material characteristics of low-borosilicate glass act as a sensitivity amplifier, severely exacerbating the conflict between the necessary extreme cooling demand and the resulting aerodynamic non-uniformity.

Finally, it is necessary to delineate the applicability boundaries of this study. The specific quantitative data—such as the exact 49.82% minimum stress uniformity or the specific polynomial coefficients describing the through-thickness stress profile—are strictly unique to this particular oval baking pan under the 30 m/s cooling condition. However, the fundamental multiphysics interaction mechanism uncovered herein (3D curvature, flow separation, HTC gradient, asynchronous shrinkage across Tg, amplified stress heterogeneity) represents a universal physical principle. This mechanism is broadly applicable to the physical tempering of any glassware featuring complex three-dimensional geometries, emphasizing that realistic aerodynamic boundaries must be explicitly resolved rather than assumed uniform.

## 4. Conclusions

In this study, a fluid–thermal–structural sequential coupling numerical simulation model was developed to investigate the tempering characteristics of curved glassware. Macroscopically validated via digital polariscope measurements, the model reasonably captured the multi-field evolution process with acceptable engineering accuracy. The main conclusions are summarized as follows:

(1) Aerodynamic Distortion and Thermal Non-uniformity: The 3D geometric curvature of the glassware fundamentally alters the cooling airflow, inducing macroscopic flow separation and localized jet stagnation. These non-uniform aerodynamic boundary conditions trigger an intense transient heat conduction process. Consequently, the temperature uniformity deviates from idealized monotonic convergence, exhibiting a unique nonlinear evolution of “destruction and subsequent reconstruction.”
(2) Stress Evolution and Spatial Distribution: The evolution of residual stress is governed by a complex chain involving temperature-dependent thermophysical transitions, non-uniform thermal gradients, and aerodynamic boundaries. After traversing the glass transition temperature, the stress uniformity experiences a severe initial drop before progressively recovering and stabilizing. Unlike flat plates with localized edge stress concentrations, the curved glassware develops a continuous, broad, spatially increasing trend of residual stress from the center to the rim.
(3) Through-Thickness Profile and Model Applicability: Macroscopically, the through-thickness residual stress maintains a stable parabolic distribution (surface compression balancing core tension), which conforms to the principles of static equilibrium. The sequential coupling strategy proposed in this work successfully captured these coupling mechanisms with a maximum relative error of less than 6%. While demonstrating high robustness and reasonable validity for rigid glass quenching, this one-way sequential framework possesses inherent applicability boundaries. Future fully two-way coupled approaches must be explored for scenarios involving extreme structural deformations, severe buckling, or material failure, where such structural changes would dynamically alter the aerodynamic boundaries.

## Figures and Tables

**Table 1:** Thermal parameters [22].

Glass Thermal Parameters	Value
Density ($\mathrm{kg}/m^{3}$)	2550
Thermal conductivity (W/m·k)	$\lambda =0.975+8.58\;\times \;10^{-4}(T+273.15)\;$
Specific heat (J/Kg⋅K)	$T\;<\;850\;K,\;C_{p}=893+0.4(T+273.15)\;-\;1.8\;\times \;10^{-7}{\left(T+273.15\right)}^{2}\;\;\;\;T\;\geq \;850\;K,\;C_{p}=1433+6.5\;\times \;10^{-3}(T+273)$

**Table 2:** Experimental measurement values for different regions.

Region	Stress (MPa)	Average Stress (MPa)
Center point	35.35	35.35
Center-Top	38.98	38.21
Center-Bottom	38.80	
Center-Left	37.28	
Center-Right	37.78	
Rim-Top	41.20	41.56
Rim-Bottom	42.16	
Rim-Left	42.02	
Rim-Right	40.84	

**Table 3:** Comparison between simulated and experimental stress values.

Region	Experimental Value (MPa)	Simulated Value (MPa)	Absolute Error (MPa)	Relative Error (%)
Center point	35.35	36.99	1.64	4.6
Inner surface	38.21	36.19	2.02	5.3
Rim region	41.56	41.81	0.35	0.8

**Table 4:** Comparison of stress at the center point.

Mesh Size	Experimental Value (MPa)	Simulated Value (MPa)	Absolute Error (MPa)	Relative Error (%)
1 mm	35.35	37.00	1.65	4.6
2 mm		36.99	1.64	4.6
5 mm		27.36	7.99	22.6
10 mm		27.31	8.04	22.7

**Table 5:** Comparison of average stress on the inner surface.

Mesh Size	Experimental Value (MPa)	Simulated Value (MPa)	Absolute Error (MPa)	Relative Error (%)
1 mm	38.21	37.21	1	2.6
2 mm		36.19	2.02	5.3
5 mm		22.83	15.38	40.3
10 mm		23.51	14.7	38.5

**Table 6:** Comparison of average stress at the rim.

Mesh Size	Experimental Value (MPa)	Simulated Value (MPa)	Absolute Error (MPa)	Relative Error (%)
1 mm	41.56	41.80	0.24	0.6
2 mm		41.81	0.25	0.6
5 mm		44.24	2.68	6.5
10 mm		39.94	1.62	3.9

## Data Availability

The original contributions presented in this study are included in the article. Further inquiries can be directed to the corresponding author.
